# Arginine 58 is indispensable for proper function of the *Francisella tularensis* subsp. *holarctica* FSC200 HU protein, and its substitution alters virulence and mediates immunity against wild-type strain

**DOI:** 10.1080/21505594.2022.2132729

**Published:** 2022-10-17

**Authors:** Pavla Pavlik, Petra Spidlova

**Affiliations:** Department of Molecular Pathology and Biology, Faculty of Military Health Sciences, University of Defence, Trebesska, Czech Republic

**Keywords:** *Francisella*, HU protein, PigR, virulence, histone-like protein, ChIP-seq, HU regulon, bacterial pathogenesis, transcription factor, nucleoid-associated protein

## Abstract

HU protein, a member of the nucleoid-associated group of proteins, is an important transcription factor in bacteria, including in the dangerous human pathogen *Francisella tularensis*. Generally, HU protein acts as a DNA sequence non-specific binding protein and it is responsible for winding of the DNA chain that leads to the separation of transcription units. Here, we identified potential HU protein binding sites using the ChIP-seq method and two possible binding motifs in *F. tularensis* subsp. *holarctica* FSC200 depending upon growth conditions. We also confirmed that FSC200 HU protein is able to introduce negative supercoiling of DNA in the presence of topoisomerase I. Next, we showed interaction of the HU protein with a DNA region upstream of the *pigR* gene and inside the *clpB* gene, suggesting possible regulation of PigR and ClpB expression. Moreover, we showed that arginine 58 and partially arginine 61 are important for HU protein’s DNA binding capacity, negative supercoiling induction by HU, and the length and winding of FSC200 chromosomal DNA. Finally, in order to verify biological function of the HU protein, we demonstrated that mutations in arginine 58, arginine 61, and serine 74 of the HU protein decrease virulence of FSC200 both *in vitro* and *in vivo* and that immunization using these mutant strains is able to protect as many as 100% of mice against wild-type challenge. Taken together, our findings deepen knowledge about the role of the HU protein in tularaemia pathogenesis and suggest that HU protein should be addressed in the context of tularaemia vaccine development.

## Introduction

*Francisella tularensis*, the aetiological agent of tularaemia, is considered to be a dangerous biological weapon [1], one which can be aerosolized to cause highly fatal pneumonic tularaemia [2]. It is known that *Francisella* is able to survive inside phagocytic cells and then escape into the cytoplasm, where it replicates massively to cause apoptosis of host cells [3]. Proteins that are believed to be responsible for the phagosomal escape and intracellular replication and that form an atypical type VI secretion system (T6SS) [4] are encoded by a gene cluster called *Francisella* pathogenicity island (FPI) [5]. The regulation of FPI genes has been well described [6–10], but the regulation of other virulence genes is more or less unknown. To date, not many transcription regulators have been identified and described in *Francisella* [11,12]. Here, we contribute to a more detailed understanding of the HU protein functioning in *F. tularensis* subsp. *holarctica* FSC200.

**Figure 1.**
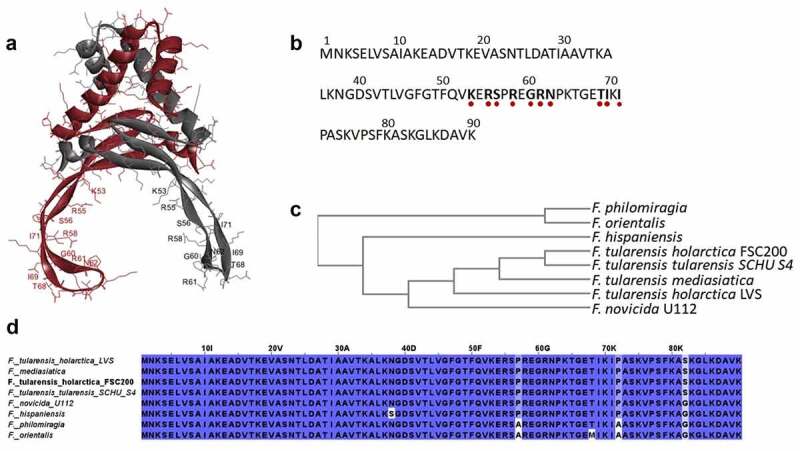
HU protein structure and phylogenetic relationship among *Francisella* species. Predicted homodimer structure of the FSC200 HU protein (FTS_0886) and highlighted amino acids that are supposed to be important for DNA binding capacity of the protein. Protein structure was predicted using HDOCK server and visualized by BIOVIA Discovery Studio Visualizer (a). FSC200 HU protein sequence with highlighted (red dots) amino acids important for DNA binding properties of the HU protein (b). Phylogram of the *Francisella* HU proteins generated using Clustal Omega (Simple Phylogeny web service) (c). Conservation of the HU protein in *Francisella* species (d).

In general, HU proteins belong to a group of nucleoid-associated proteins that often are referred to as histone-like proteins for their functional similarity with eukaryotic histones [13,14]. The pleiotropic role of the HU protein has been well documented in *Escherichia coli* [15–19], and its association with the expression of virulence genes in several bacterial species [20–22] and viruses [23–25] also has been reported. HU proteins are highly conserved among bacterial species, so it is assumed that they play similar roles. They are primarily DNA structuring proteins, but their functioning as transcription factors also has been shown [26]. Apart from its intracellular role, secretion of the HU protein and its extracellular role also have been studied [27]. HU protein exists as both homo- [28] and hetero-dimer [29]. In *Francisella*, HU protein is encoded by a single *hupB* gene and exists as a homodimer [20]. It consists of 90 amino acids, and its N-terminal and C-terminal parts are responsible for dimer assembly. The inner part, consisting of several amino acids, is believed to be important for DNA binding capacity of the HU protein [30] (Figure 1a,b). HU protein is highly conserved also among *Francisella* species (Figure 1c,d). Recently, we have shown the involvement of HU protein in FSC200 virulence and a huge impact of *hupB* gene deletion on the FSC200 proteome, whereby the level of almost one-quarter of all proteins was changed [20]. We showed that *hupB* deletion led to decreased production of FPI proteins and of their regulatory protein PigR on both transcription and protein levels [20]. Here, we investigated whether HU protein can control *pigR* on the gene level.

PigR is one of the transcription factors in *Francisella* spp. affecting virulence of the bacterium [12]. PigR, also termed FevR protein, is a necessary component of the PigR/MglA/SspA complex that regulates virulence gene expression in *Francisella* spp. [10,31–33] and thus is necessary for bacterial replication [10]. We also investigated HU protein’s further molecular functions and key amino acids relating to its main functions.

Moreover, we investigated the interaction of HU protein with *clpB*, which is one of the ChIP-seq-identified regions. Having many functions, chaperone ClpB is a highly conserved protein among bacterial species. It has been implicated in various stress responses and virulence [34]. ClpB plays an important role in *Francisella* T6SS assembly and intracellular replication [35,36], and it has been suggested as a good target for therapeutic treatment [34]. Elucidation of its regulatory protein may contribute to future research of ClpB, and we suggest that HU protein could regulate this chaperone on the gene level.

## Materials and methods

### Bacterial strains and growth conditions

Bacterial strains used in this study are summarized in Table 1. These strains were cultured as we have described previously [20].

**Table 1.** Bacterial strains used in this study.

Bacterial strain	Description	ATB	Source
*Francisella tularensis* subsp. *holarctica* FSC200	Wild type strain	-	*Francisella* Strain Collection (FSC) of the Swedish Defence Research Agency, Umea, Sweden
*Francisella tularensis* subsp. *holarctica* FSC200/ΔHU	Deletion mutant strain lacking the *hupB* gene (coding for HU protein)	-	[20]
*Francisella tularensis* subsp. *holarctica* FSC200/HU_HA	Deletion mutant strain complemented *in trans* using pKK289GFP plasmid, HU protein is tagged with a HA tag	Km	This study
*Francisella tularensis* subsp. *holarctica* FSC200/HU_HA/R58Q	Deletion mutant strain complemented *in trans* using pKK289GFP plasmid, HU protein is mutated tagged with a HA tag, R58 is mutated to the Q	Km	This study
*Francisella tularensis* subsp. *holarctica* FSC200/HU_HA/R61Q	Deletion mutant strain complemented *in trans* using pKK289GFP plasmid, HU protein is tagged with a HA tag, R61 is mutated to the Q	Km	This study
*Francisella tularensis* subsp. *holarctica* FSC200/HU_HA/S74A	Deletion mutant strain complemented *in trans* using pKK289GFP plasmid, HU protein is tagged with a HA tag, S74 is mutated to the A	Km	This study

### *Complementation of FSC200/HU_HA* in trans

The *F. tularensis* subsp. *holarctica* FSC200 *hupB* gene containing HA tag (human influenza haemagglutinin) was amplified by PCR using pKK_0886_F and pKK_0886_R_HA primers (Table S1). Final construct of shuttle vector pKK289KmGFP containing *hupB*-HA was prepared by a method as we described previously [20] and it was electroporated into *F. tularensis* subsp. *holarctica* FSC200/∆HU [37]. The complemented mutant was named as *F. tularensis* subsp. *holarctica* FSC200/HU_HA.

### Site-direct mutagenesis

The point mutations were introduced using QuikChange II Site-Directed Mutagenesis kit (Agilent, 200523), mutagenic primers (Table S1), and vector pKK289 Km *hupB-*HA as a template. Insertion of mutations was verified by sequencing (Institute of Microbiology, Czech Academy of Sciences, Prague). The electroporation was used to introduce the constructs with desired mutations into *F. tularensis* subsp. *holarctica* FSC200/∆HU [37], and the resulting strains were denoted as *F. tularensis* subsp. *holarctica* FSC200/HU_HA/R58Q, FSC200/HU_HA/R61Q, and FSC200/HU_HA/S74A.

### Growth curves

Overnight cultures of bacterial strains (FSC200, FSC200/ΔHU, FSC200/HU_HA/R58Q, FSC200/HU_HA/R61Q, and FSC200/HU_HA/S74A) were diluted to final optical density at 600 nm of 0.1 in complete Chamberlain’s medium. Optical densities were measured in a 96-well plate in pentaplicates at 37°C overnight. The growth kinetics were determined using a FLUOstar Optima microplate reader (BMG Labtech, Offenburg, Germany). The experiment was repeated three times. One-way ANOVA followed by Bonferroni’s multiple comparison test was used for statistical analysis (GraphPad Prism version 5).

### Purification of FtHU

HU_HA or proteins with point mutations were purified from the corresponding strains’ lysates in Tris-buffered saline (TBS) obtained from the overnight-cultured bacterial suspensions. Proteins were purified using Pierce Anti-HA Agarose Beads (Thermo Fisher Scientific, 26181) according to the manufacturer’s instructions. Proteins were eluted by 3 M NaSCN (sodium thiocyanate). Buffer exchange was then done using Zeba™ Spin Desalting Columns (Thermo Fisher Scientific, 89890). Protein concentrations were calculated by BCA assay (Thermo Fisher Scientific, 23225).

### Chromatin immunoprecipitation and NGS sequencing (ChIP-seq)

#### ChIP sample preparation

Bacterial strains (FSC200/HU_HA, FSC200/∆HU) were grown overnight in Chamberlain’s medium with appropriate antibiotic (kanamycin for FSC200/HU_HA; 20 µg/mL) at 37°C and 200 rpm. Samples were subsequently diluted to optical density at 600 nm of 0.1. The cultures were incubated at 37°C and 200 rpm to reach final optical density of 0.25–0.40 (early exponential growth phase) or 0.7–0.8 (late exponential growth phase). In the case of stress growth conditions, hydrogen peroxide (Sigma Aldrich, H1009) at 0.03% final concentration was added to induce oxidative stress conditions and cultures were stressed for 1 h (only for the early exponential growth phase). Formaldehyde (Sigma Aldrich, 252549) at final concentration of 1% was added to crosslink protein–DNA complexes. The cultures were incubated for 20 min at room temperature and 80 rpm, after which 0.5 M Tris was used for quenching the formaldehyde. The cultures were then incubated for 20 min at 4°C and 80 rpm and centrifuged. Bacterial pellets were washed by TBS three times and resuspended in 1 mL TBS containing protease inhibitor (cOmplete™ Protease Inhibitor Cocktail, Roche, 000000011836145001). Bacterial cell lysates were prepared by French press (16 000 psi). Suspensions were sonicated (50% amplitude, 10 s on/20 s off, 140 cycles) to achieve DNA fragment size of about 500–600 bp. Immunoprecipitation of HU–DNA complexes was performed using Pierce Anti-HA Agarose Beads (Thermo Fisher Scientific, 26182) and Pierce Spin Column-Screw Cap (Thermo Fisher Scientific, 69705). The samples were undergoing reverse crosslink at 65°C, overnight. Samples were then treated with Proteinase K (Thermo Fisher Scientific, AM2548) and RNase A (Thermo Fisher Scientific, R1253). DNA was cleaned and concentrated using Nucleospin gDNA Clean-up Kit (Macherey Nagel, 1703/003).

Sequencing libraries were prepared using NEBNext Ultra II DNA Library Prep Kit for Illumina (cat. no. E7645 L). Quality control of libraries was performed using Agilent Bioanalyzer 2100 High Sensitivity DNA Kit (cat. no. 5067–4626). MiSeq Reagent Kit v2 (MS-102-2003) was used for MiSeq sequencing. Sequencing libraries and NGS sequencing on the MiSeq Illumina platform were carried out at the Institute of Molecular Genetics, Czech Academy of Science in Prague, Czech Republic.

#### ChIP data evaluation

Quality of raw reads was first evaluated using FastQC (v0.11.5) and MultiQC (v1.7) tools. Poor-quality reads were then trimmed or removed from the data set using cutadapt (v1.18). The adjusted reads were aligned with Bowtie2 (v2.2.6) against the reference genome of *Francisella tularensis* subsp. *holarctica* FSC200 (NCBI genome database). The aligned reads were converted and sorted using SAMtools (v1.7) and duplicate reads were removed using Picard Tools (v1.95). Alignment statistics were created using BAMtools (v2.4.0) and qualimap (v2.2.1). The DNA binding sites (peaks) were predicted using MACS2 (v2.1.2). Due to the small size of the sample data, MACS2 was run with the ´–nomodel´ settings, and the size of the genome was set to 1.89e + 06 bp, ´–extsize´ fix-sized fragments 147 bp, and *p*-value cut-off to 0.05. The called peaks were annotated based upon the gff3 annotation file using BEDtools (v2.27.1).

Data analyses were carried out at SEQme s.r.o., Dobris, Czech Republic.

#### Binding motifs identification

The motifs in a group of related DNA were determined using the Multiple EM for Motif Elicitation (MEME-ChIP) tool (v5.0.2).

### Electrophoretic mobility shift assay (EMSA)

Interaction of the HU protein with DNA was analysed by the EMSA method. DNA Retardation Gels (6%) (Invitrogen, EC6365BOX) were used for HU–DNA complex analysis. The different amounts of the HU protein (0–800 ng) were incubated with 100 ng of DNA corresponding to the 477 bp sequence upstream of the *pigR* gene that was obtained using PCR amplification according to the FSC200 template. Reaction mixtures were incubated in binding buffer (20 mM Tris-HCl, pH 8, 0.1 mM EDTA-Na_2_, 50 mM KCl, 10 μg/mL bovine serum albumin (BSA), 5% glycerol, 0.1 mM DTT, 0.05% Brij 58) for 20 min at 4°C. The samples were loaded onto the gel and underwent electrophoresis (0.5x TBE, 100 V, 160 min). The complexes were visualized using UV light and SYBR®Safe DNA gel stain (Invitrogen, S33102). Interactions of mutant HU protein forms with sequence upstream of the *pigR* or 568 bp sequence corresponding to *clpB* (DNA region identified in ChIP-seq analysis obtained using PCR according to the FSC200 template) were analysed using 1% TBE agarose gel. Sixty nanograms of DNA and 500 ng of proteins were used. The mixtures underwent the same process as described above.

### DNA supercoiling assay

HU_HA protein and its mutant variants were tested to introduce negative DNA supercoils in the presence of human topoisomerase I (Sigma-Aldrich, T9069–250 UN). Briefly, the negatively supercoiled pBluescript KS− (100 ng) was incubated in the presence of human topoisomerase I (4 U), and various amounts of wild-type (WT) or mutant HU_HA proteins (0, 187.5, 375, 750, 1500 ng) were added in a 50 µL reaction containing an assay buffer (10 mM Tris-HCl, pH 7.9, 150 mM NaCl, 0.1% BSA, 5% glycerol) for 90 min at 37°C. The reaction was stopped by adding 0.33 M NaOAc. The DNA was then ethanol precipitated and resuspended in 10 mM Tris-HCl, pH 7.9. The samples were loaded onto the 1% gel, resolved by electrophoresis (1x TAE, 50 V, 180 min), stained using SYBR®Safe DNA gel stain (Invitrogen, S33102), and then visualized by UV light.

### *Semi-quantitative analysis of* pigR *expression in mutant strains (RT-PCR)*

RNA from bacterial strains (FSC200, FSC200/ΔHU, FSC200/HU_HA/R58Q, FSC200/HU_HA/R61Q, and FSC200/HU_HA/S74A) were isolated using RNeasy Mini Kit (Qiagen, 74106) according to the manufacturer’s instructions. QuantiTect®Reverse Transcription Kit (Qiagen, 205311) was used for genomic DNA elimination and reverse transcription according to the manufacturer’s instructions. cDNA was used for PCR amplification of *pigR* and *rpoA* using appropriate primers (Table S1).

### Atomic force microscopy

#### HU–DNA complexes

Atomic force microscopy (AFM) was used for visualization of HU–DNA complexes. The samples were prepared in the same way as were samples for the ChIP experiment. The samples of FSC200/HU_HA in early exponential (OD_600_ 0.4) and late exponential growth phases (OD_600_ 0.8) were compared. Two different sets of growth conditions were used for AFM analysis, as well, these being standard and oxidative stress growth environments. The FSC200/ΔHU was used as a negative control. Purification of the final eluate was performed using ZEBA Spin Desalting Columns (Thermo Fisher Scientific, 89893) and exchange buffer (20 mM Tris, pH 8.0, 50 mM NaCl, 5 µM EDTA, 1.2% glycerol).

#### HU-chromosomal DNA

Chromosomal DNA (chDNA) was isolated from FSC200 and FSC200/ΔHU strains using the DNeasy Blood & Tissue Kit (Qiagen, 69504) according to the manufacturer’s instructions. Samples of chDNA and different variants of HU protein were prepared in binding buffer (20 mM Tris-HCl, pH 8.0, 0.1 mM EDTA-Na_2_, 50 mM KCl, 5% glycerol, 0.1 mM DTT, 0.05% Brij 58) in a molar ratio of 1:20, where protein molarity corresponds to 100 fmol.

#### AFM visualization

Visualization of DNA fragments was performed in air with mica as the substrate. Initial samples were diluted 20 × in HEPES buffer with Mg^2+^ ions (40 mM HEPES, 10 mM MgCl_2_, pH 7.0). DNA fragments (20 µL) were adsorbed on a freshly cleaved mica surface (SPI Supplies, West Chester, PA, USA) at room temperature for 30 min. Afterwards, the surface was rinsed with distilled water and dried with compressed air to prevent undesired drying of the buffer salts. The AFM scanning was performed using Dimension FastScan Bio (Bruker, USA) in PeakForce Tapping mode. A ScanAsyst-Air probe (Bruker, USA) with a spring constant k of 0.4 N/m was used. The AFM images were processed using the Gwyddion software package. The AFM images were processed in FiberApp to measure lengths of the DNA strands.

*In vitro* proliferation in bone marrow-derived macrophages

*In vitro* proliferation of the strains with modified HU protein (R58Q, R61Q, and S74A) was performed as we have described previously [20,38].

### Infection of the mouse model

The ability of bacterial strains FSC200, FSC200/HU_HA/R58Q, FSC200/HU_HA/R61Q, and FSC200/HU_HA/S74A to cause tularaemia in mice was studied, as we described previously [20]. Groups of 10 mice were infected subcutaneously (s.c.) with 200 μL of bacterial suspension with infection doses of 10^2^ CFU/mouse. Mice were observed for 30 d, and any deaths were noted. All mice survived except those infected with strain FSC200. The survivors were challenged 30 days post-infection with 10^2^ CFU/mouse (s.c.) of the FSC200 to study the ability of the mutant strains to protect mice against WT challenge.

### Statistical analysis

Statistical significances were analysed using GraphPad Prism version 5 (GraphPad Software, La Jolla, CA, USA). The specific statistical test types used for each experiment are indicated in the figure captions.

## Results

### FSC200 HU protein interaction with the genome

#### In silico determination of HU binding sites throughout the whole genome

To identify the HU protein binding sites in the FSC200 genome, ChIP-seq was performed using FSC200/HU_HA along with FSC200/ΔHU as a negative control. Sequences of eight samples (*F. tularensis*) belonging to two groups according to cultivation conditions used (hereinafter termed “Standard” and “Stressed”) were analysed. First, the data were evaluated for base-calling quality, number of duplicated sequences, and adapter sequences. Between 0.2% and 1.7% of reads were trimmed for various reasons. The adjusted reads were aligned with the reference genome sequence (*Francisella tularensis* subsp. *holarctica* FSC200). The duplicated reads were removed using Picard Tools. The final numbers of duplicated reads were very low (<0.021065%). Reads across all samples were mapped to a reference genome with success exceeding 94%. The correlation of samples within a group corresponded to the assumption that control samples from both groups differ significantly from target samples. The control sample in the Stressed group was significantly different from that in the group under Standard conditions. Ninety-two percent of the reference sequence was covered at least 30 times. The median insert size was comparable among all samples (623–678 nt). The DNA binding sites (peaks) were predicted using MACS2 tools, where the control samples were used like mock data (background level data). Due to the small amount of sample data, it was necessary to change the default settings, especially for the Stressed group. The MACS2 detected 261 binding sites referring to the Standard group data set and 105 for the Stressed group data set (Table S2, available at: https://docs.google.com/spreadsheets/d/1wAT9OcUTomvDJudhI5963gF-rS3JwsZp/edit#gid=537570250). Almost all peaks were successfully annotated except for two peaks in the Standard group and one peak in the Stressed group. No overlap was identified between peaks of the two groups. Selected interesting genes, containing HU protein binding site under standard or stress growth conditions, are summarized in Table 2. Among HU protein binding sites under both standard and stress growth conditions, for example, different sequences from the *clpB* gene were found, thus suggesting that HU protein may somehow participate in regulation of this important chaperone protein in FSC200 under various growth conditions. We also found a potential HU protein binding site inside the *iglB* gene, which is one of the FPI genes, under standard growth conditions. Many of the detected standard binding sites are sequences belonging to genes involved in ordinary metabolic processes within the cell, such as *polA* (DNA polymerase I), *ftsA* (cell division protein FtsA), *dnaB* (replicative DNA helicase), and *rpoH* (RNA polymerase factor sigma-32). Among the HU protein binding sites identified under stress conditions many sites were found inside genes that are involved in stress response and increased ATP consumption, such as *fadE* (Acyl-CoA dehydrogenase), *nuoDFGL* (NADH dehydrogenase subunits), and *atpBDF* (ATP synthase subunits) (Table S2, available at: https://docs.google.com/spreadsheets/d/1wAT9OcUTomvDJudhI5963gF-rS3JwsZp/edit#gid=537570250).

**Table 2.** Selected genes containing HU protein binding site under standard od stress growth conditions.

Name	gene	HU binding site under standard or stress conditions	biological function
Chaperone protein ClpB	*clpB*	standard/stress	virulence
Intracellular growth locus B	*iglB*	standard	virulence
DNA polymerase I	*polA*	standard	metabolism
Cell division protein FtsA	*ftsA*	standard	cell division
Replicative DNA helicase	*dnaB*	standard	DNA
RNA polymerase factor sigma-32	*rpoH*	standard	heat shock response
Acyl-CoA dehydrogenase	*fadE*	stress	metabolism, energy of cell
NADH dehydrogenase subunits	*nuoDFGL*	stress	energy of cell
ATP synthase subunits	*atpBDF*	stress	energy of cell

#### Potential HU protein binding motifs identification

**Figure 2.**
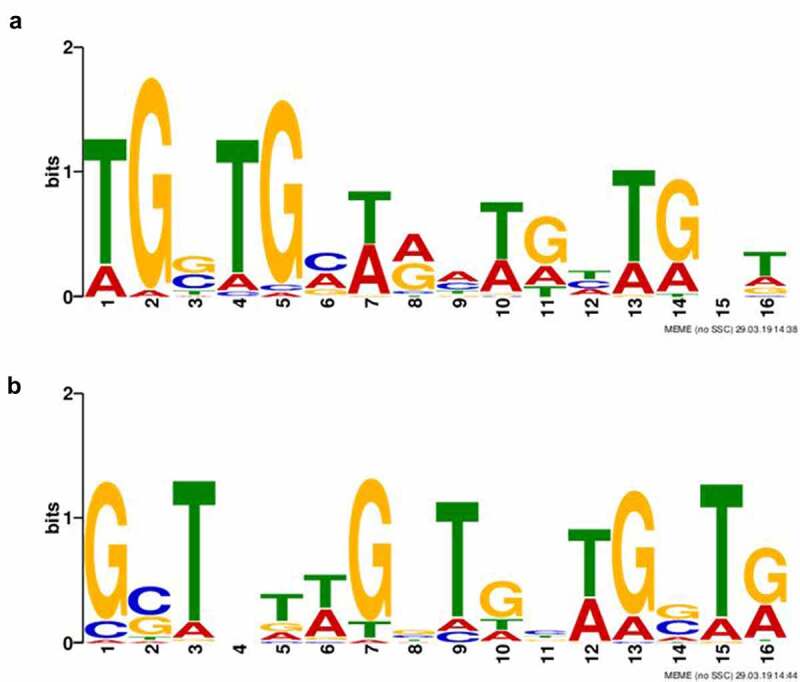
Identification of HU protein binding motifs. Two different HU protein binding motifs depending upon growth conditions ((a) standard growth conditions and (b) oxidative stress conditions) were found using the Multiple EM for Motif Elicitation (MEME-ChIP) tool (v5.0.2). Binding motifs were found in all target sequences identified using ChIP-seq.

As the ChIP-seq approach revealed >200 and >100 binding sites under standard or oxidative stress growth conditions, respectively, we endeavoured to find out if these binding sites are sequence non-specific or characterized by conserved binding motifs. Based upon ChIP-seq narrow peaks for each group (Standard and Stressed), sequence motifs were identified separately. Just one binding motif was discovered for each group. The binding motifs were found in all identified target sequences. Both 16 bp in length, the binding motifs are shown in Figure 2.

#### Imaging of HU–DNA complexes

**Figure 3.**
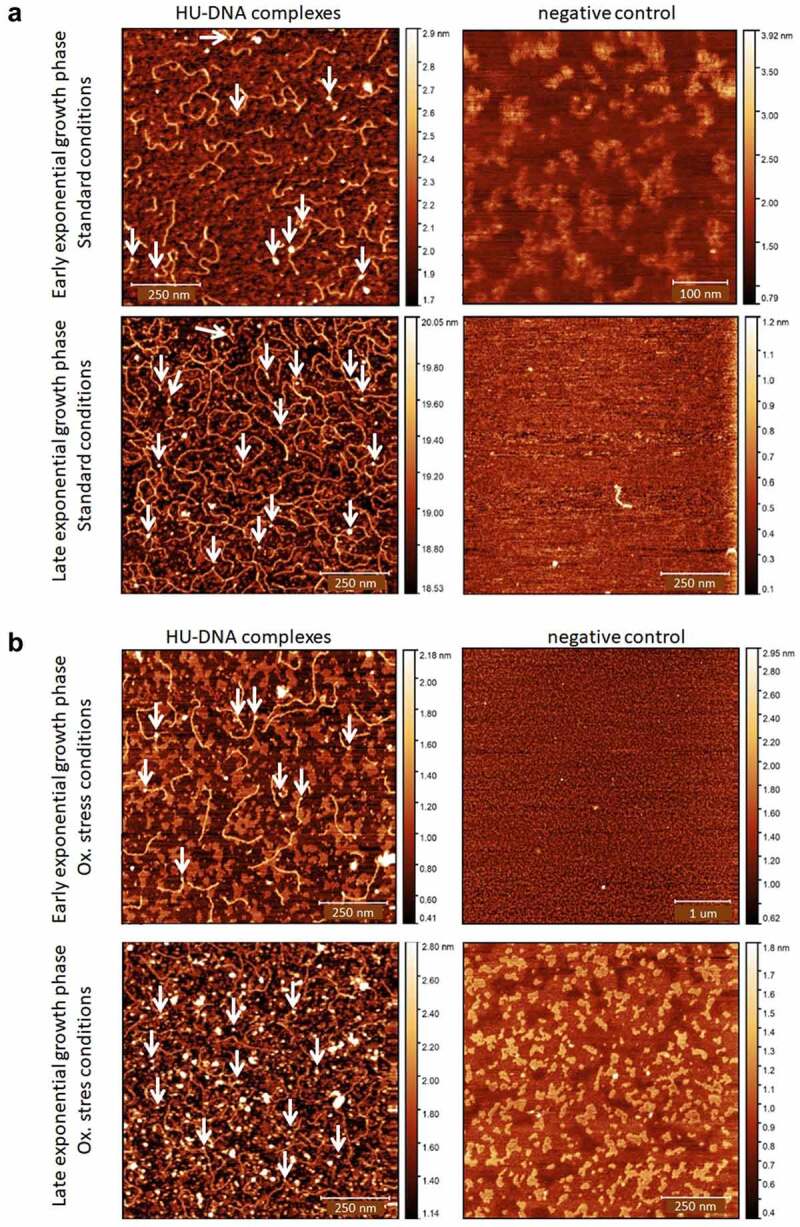
Visualization of HU–DNA complexes using AFM. *F. tularensis* FSC200/HU_HA was grown under standard (a) or oxidative stress (b) conditions. AFM imaging was performed in PeakForce Tapping mode. Samples purified from *F. tularensis* FSC200/ΔHU were used as negative controls. Larger quantities of DNA and HU–DNA complexes (HU protein corresponds to white dots) were identified in the late exponential phase. Imaging of samples of negative control confirmed the absence of HU protein, DNA, and their complexes, as well. The height profile scale is shown on the right.

The AFM nanoimaging technique was used for the visualization of HU–DNA samples. Analysed samples were prepared by the same procedure as were samples for the ChIP-seq experiment (HA-precipitated HU–DNA complexes). HU–DNA complexes obtained during early and late exponential growth phases were compared. FSC200/ΔHU mutant strain was used as a negative control. Imaging of samples was successful, and parts of DNA with bound proteins were visualized. As expected, larger quantities of DNA were identified in the late exponential phase in comparison with the early exponential phase (Figure 3). Imaging of negative control samples then confirmed the absence of HU protein, DNA, and their complexes.

### *HU protein is able to bind upstream of the* pigR *gene*

**Figure 4.**
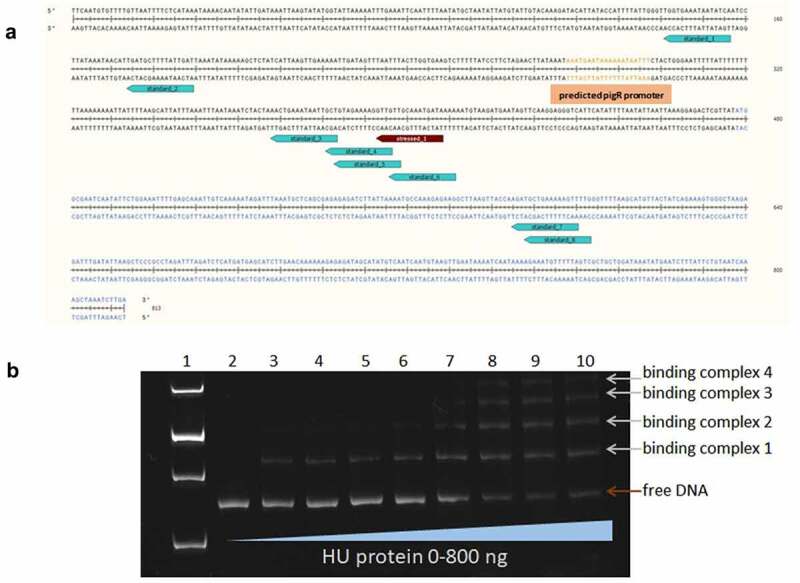
Identification of HU protein binding motifs within the sequences of the 477 bp upstream of the *pigR* gene and *pigR* gene. (a) in silico analysis of DNA sequence upstream of the *pigR* gene (in black) and within *pigR* gene (in blue) showed 8 possible binding sites (turquoise) of the HU protein for standard (6 of them upstream of the *pigR* gene and two within the gene) and one binding site (red) for oxidative stress conditions. (b). EMSA experiment confirmed binding of the HU protein to 477 bp DNA sequence upstream of the *pigR* gene. 100 ng of 477 bp DNA sequence upstream of the *pigR* gene was added to a various amounts of HU protein (0-800 ng) in a binding buffer and incubated for 20 min on ice. Then, the samples were resolved on DNA Retardation Gels (6%) (Invitrogen, EC6365BOX). There is a strong correlation between the amount of the HU protein and the decrease of free DNA and the increased number of HU–DNA complexes. 1- standard (AccuBand 100 bp DNA Marker II, SMOB-DM2000), 2- control sample containing the DNA fragment in a binding buffer without any protein, 3–10 – samples containing the DNA fragment in a binding buffer with increasing amounts of HU protein. Free DNA and HU–DNA binding complexes are depicted by red and white arrows respectively.

Based upon our previous study [20], where PigR, one of the main virulence regulators in *Francisella*, was identified as a protein with reduced expression on transcriptional and protein levels in the HU deletion mutant strain, we examined whether transcription factor HU can regulate expression of the *pigR* gene even though neither that gene nor its regulatory region was detected in ChIP-seq analysis. The presence of a potential HU binding motif in the FSC200 strain genome was analysed using the bioinformatics tool FIMO online software (v5.0.5) with a *p*-value of 0.001. Overall, 20399 Standard and 14,882 Stressed binding motifs were found through the FSC200 genome (Table S3, available at: https://docs.google.com/spreadsheets/d/1Qm5AVHQB9DMnKIo7xzYQ6rhm8JZEP-z1/edit#gid=362571185). Altogether six possible Standard binding sites and one Stressed site of HU protein were found in a region 477 bp upstream of the *pigR* gene (Figure 4a). Two possible standard binding sites were identified within the *pigR* gene (Figure 4a). Given that some of these sites overlapped, we cannot rule out that only some of these sites can be occupied at the same time. This idea was confirmed using the EMSA method, whereby we showed that under our conditions HU protein binds the DNA fragment corresponding to 477 bp upstream of the *pigR* gene four times (Figure 4b). This finding proves not only a possible role of HU protein in *pigR* gene regulation but also contributes to verification of the identified binding motifs of HU protein.

### *Arginine 58 is a key amino acid for HU protein DNA binding capacity to the region upstream of the* pigR *gene*

**Figure 5.**
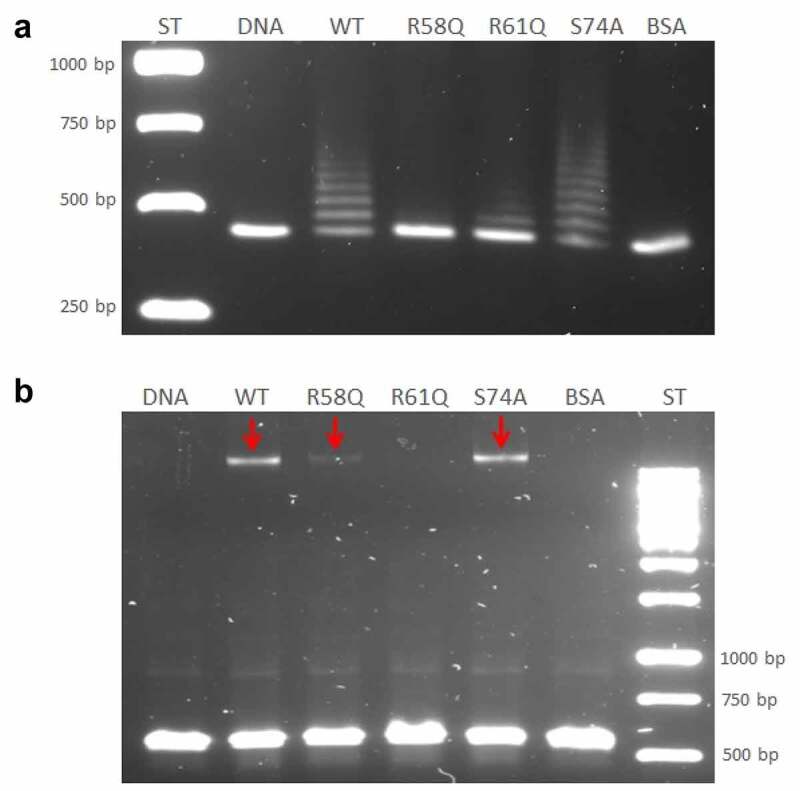
Arginine 58 and arginine 61 are essential for the DNA binding capacity of HU protein. The role of R58, R61, and S74 on DNA binding capacity in comparison with the WT form of HU_HA protein was tested using EMSA. 500 ng of WT HU_HA protein or HU protein mutant variants were mixed with 60 ng of (a) 477 bp DNA sequence upstream of the *pigR* gene or (b) 568 bp DNA sequence corresponding to the part of *clpB* gene (identified in ChIP-seq analysis) in a binding buffer for 20 min on ice and resolved on 1% TBE/agarose gel. ST – molecular weight standard (GeneRuler 1 kb DNA Ladder, Thermo, SM0311), DNA – control sample containing the DNA fragment in a binding buffer without any protein, WT, R58Q, R61Q, S74A, and BSA – samples containing the DNA fragment in a binding buffer with the appropriate protein.

Further study regarding the HU protein binding capacity was conducted. Based upon bioinformatic analysis of evolutionarily conserved amino acids of HU protein important for the binding capacity of HU protein in other bacteria [26], several mutant strains with point mutations were created, as follows: R58Q, R61Q, and S74A. The ability of mutant HU proteins to bind DNA corresponding to the 477 bp upstream of the *pigR* gene was tested using the EMSA method. WT HU protein purified from FSC200/HU_HA strain and BSA was used as positive and negative controls, respectively. The R58 mutation showed the strongest effect on the ability of the HU protein to bind to DNA compared to other mutated amino acids and the WT HU protein (Figure 5a). The R58 mutation abolished the DNA binding capacity of the HU protein. Probably no HU/R58Q-DNA complex had been formed and DNA fragment migrated through the agarose gel comparably to the control DNA sample and the sample from the negative control reaction with BSA. The other protein variants formed protein–DNA complexes but with different efficiency. The WT form of HU protein, as well as the HU/S74A mutant protein, formed several protein–DNA complexes, and the HU/S74A mutant protein seems to form more complexes than does the WT HU protein. Mutation in R61, on the other hand, resulted in diminished DNA binding capacity, and many fewer protein–DNA complexes were visible on agarose gel. This demonstrates an important role of R61, as well, in DNA binding capacity (Figure 5a). This result confirmed the ability of HU protein to bind to the DNA region upstream of the *pigR* gene and showed the importance (mainly in position 58) of conserved arginines within this process.

### *HU protein binds inside the* clpB *gene*

We used the EMSA method to verify HU protein binding regions and binding motifs that had been identified in ChIP-seq analysis. A part of the *clpB* gene was chosen that is one of the sites containing potential binding motifs under both standard and stress growth conditions. We proved that HU protein binds in this region and forms a complex with a part of the *clpB* gene (Figure 5b), suggesting HU protein involvement in ClpB regulation on the gene level. As expected, we identified only one HU–DNA complex (red arrows in Figure 5b). We also tested the importance of R58, R61, and S74 amino acids for the HU protein DNA binding capacity in *clpB*. WT HU protein and BSA, respectively, were used as positive and negative controls. The R61 mutation showed the greatest effect on the ability of the HU protein to bind to *clpB* (no complex formed) and the R58 mutation showed some effect (decreased amount of DNA in complex) compared to S74 and the WT HU protein (Figure 5b).

### *Alteration in the arginine 58 of the HU protein leads to* pigR *downregulation on the transcriptional level*

**Figure 6.**
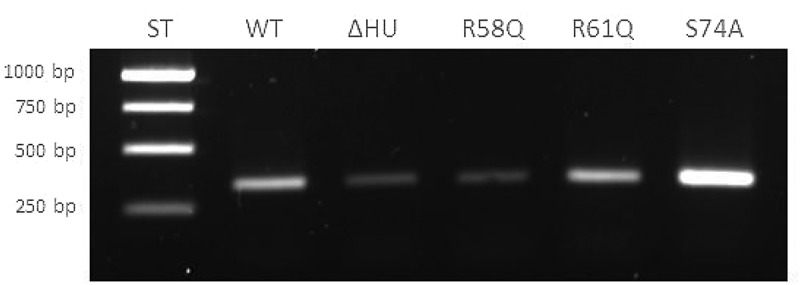
Semi-quantitative RT-PCR demonstrates decreased transcription level of *pigR* gene in FSC200/ΔHU and FSC200/HU_HA/R58Q. Expression of *pigR* in all tested strains was verified on transcription level using reverse transcription followed by PCR. Samples were analysed by gel electrophoresis. *pigR* showed significantly decreased expression in deletion mutant strain and FSC200/HU_HA/R58Q in contrast to WT. This result suggests HU protein participation in *pigR* regulation.

To confirm the results described above and to show the importance of R58 in the regulation of *pigR* expression, we performed reverse transcription-PCR (RT-PCR) of *pigR* using RNA isolated from all tested strains (FSC200, FCS200/ΔHU, FSC200/HU_HA/R58Q, FSC200/HU_HA/R61Q, and FSC200/HU_HA/S74A). The transcription level of *rpoA* was chosen as a control (not shown) inasmuch as the protein level of RpoA was not altered in deletion mutant strain FSC200/ΔHU [20]. Consistent with our expectation and our previous experiment [20], the *pigR* (Figure 6) showed significantly decreased expression in deletion mutant strain FSC200/ΔHU in contrast to WT. Moreover, this experiment proved the necessity of R58 of FSC200 HU protein in *pigR* expression, whereas the alteration of arginine in position 61 had no effect. Surprisingly, the transcription level of *pigR* in FSC200/HU_HA/S74A increased in comparison to that in WT. Taken together, these results confirm a role of HU protein in *pigR* expression.

### F. tularensis *FSC200 HU protein is able to cause negative supercoiling of DNA in the presence of human topoisomerase I, and R58 is key to this process*

**Figure 7.**
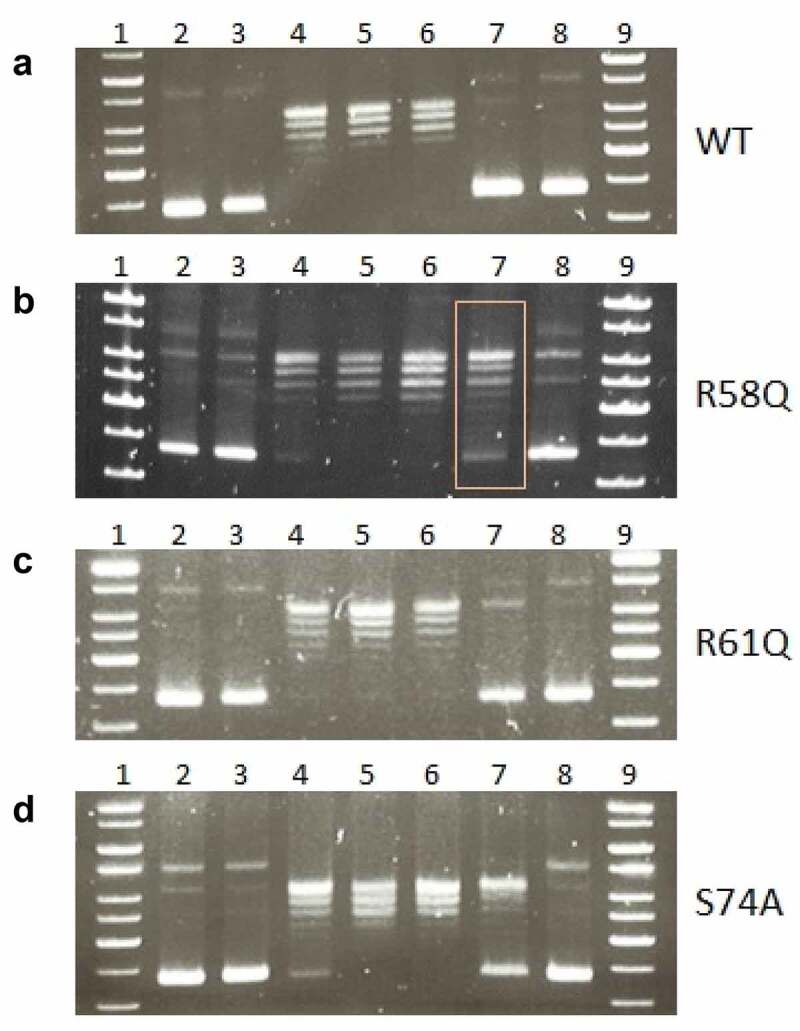
Mutation in arginine 58 affects the ability of HU protein to introduce negative DNA supercoiling. Effects of R58 (b), R61(c), and S74 (d) mutations on the ability of HU protein to introduce negative DNA supercoiling in the presence of human topoisomerase I in comparison to the WT form of HU protein (a) was investigated. 1, 9 - molecular weight standard (GeneRuler 1 kb DNA Ladder, Thermo, SM0311), 2 - supercoiled pBluescript KS- DNA incubated in an assay buffer without any protein, 3 - supercoiled pBluescript KS- DNA incubated in an assay buffer with 1500 ng of appropriate HU protein and no human topoisomerase I, 4–8 - supercoiled pBluescript KS- DNA incubated in an assay buffer with 4 U of human topoisomerase I and the increasing amount of corresponding HU proteins (0, 187.5, 375, 750, and 1500 ng).

HU protein is known to act in a manner opposite that of topoisomerase I, and here we investigated the ability of FSC200 HU protein to introduce negative supercoiling of DNA in the presence of human topoisomerase I. We verified that the WT form of HU protein (HU_HA) has this function (Figure 7a) and we tested the HU protein variants for this capacity. Mutations in R58, R61, and S74 were investigated. We identified the importance of R58 (Figure 7b) rather than of either R61 (Figure 7c) or S74 (Figure 7d). Similarly, just as R58 is important for the DNA binding capacity of HU protein, as we have shown above, R58 also is necessary for the full function of HU protein in negative supercoiling induction.

### Coiling and length of chDNA are dependent upon HU protein

**Figure 8.**
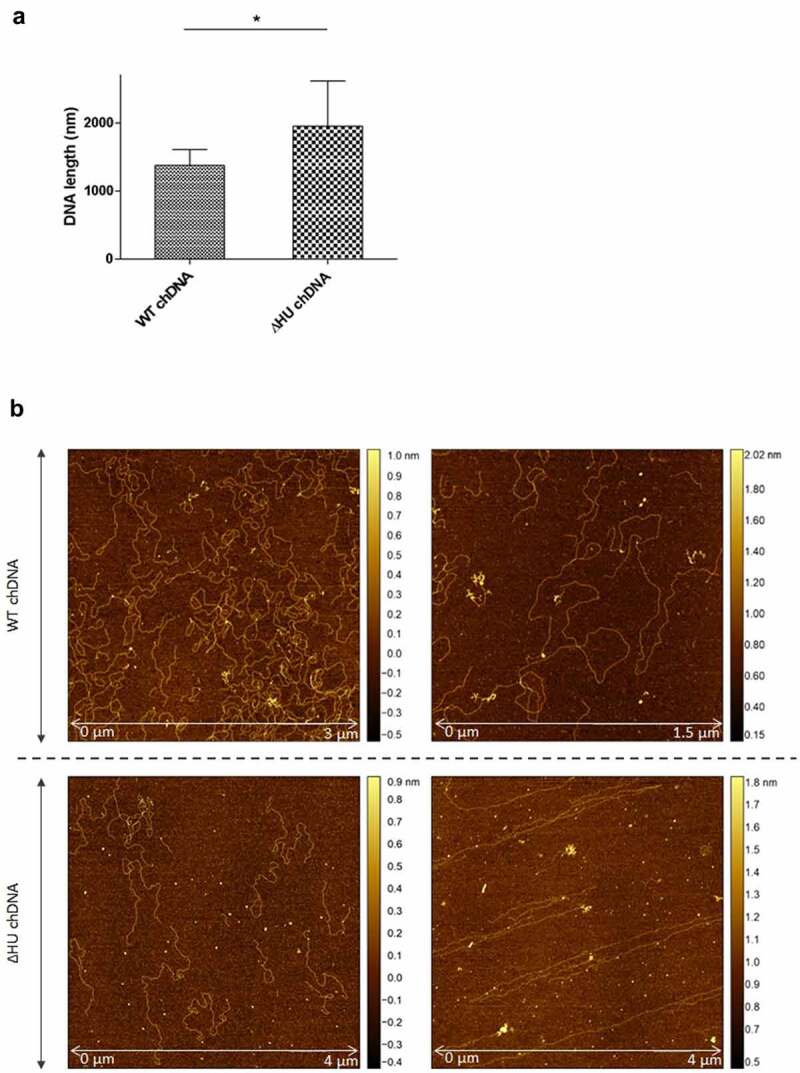
AFM images of ΔHU chDNA in the absence/presence of WT and mutant HU protein variants. Comparison of ΔHU chDNA compactness changes depending upon HU protein variant addition. Two representative images (different scale) of FSC200/ΔHU strain chDNA combined with HU protein variants are displayed in each row. The height profile scale is shown on the right.

To visualize the effect of HU protein on the shape of *F. tularensis* subsp. *holarctica* chDNA, we performed AFM imaging of chDNA isolated from either FSC200 or FSC200/ΔHU (that means in the presence or absence of the HU protein). The AFM images were processed in FiberApp to measure the length of the DNA strands, and we observed significant differences. chDNA isolated from the strain lacking the HU protein was more relaxed, and long straight parts were observed, whereas chDNA isolated from the WT strain was ordinarily coiled (Figure 8a,b).

**Figure 9.**
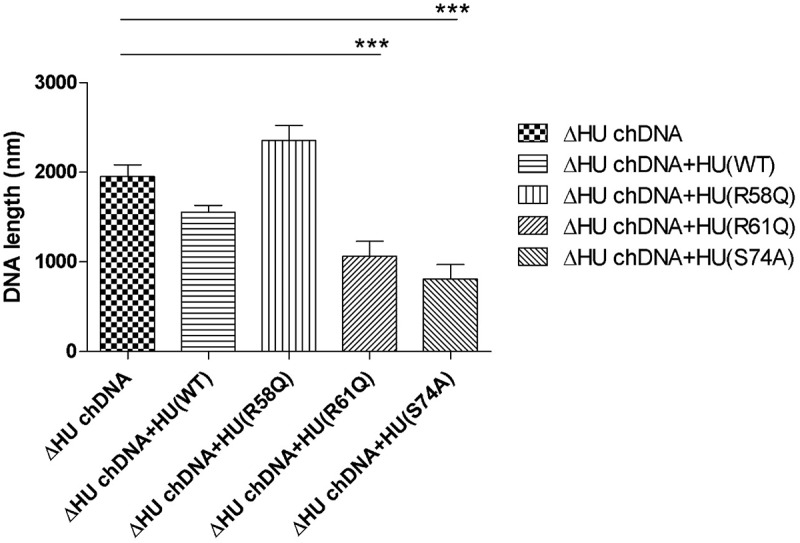
Intracellular replication of *F. tularensis* FSC200 and mutant strains with point mutations in *hupB* gene inside BMMs. Macrophages were infected with FSC200 or tested mutant strains at MOI of 50 and the numbers of replicating bacteria expressed as CFU counts were determined at 1, 6, 24, and 48 h post infection. Error bars represent the mean ± sd derived from three independent experiments. Statistical significance was analysed using Two-way ANOVA followed by Bonferroni’s multiple comparison test. *P*-value <0.05 *, P < 0.01 **, P < 0.001 ***, ns not significant.

**Figure 10.**
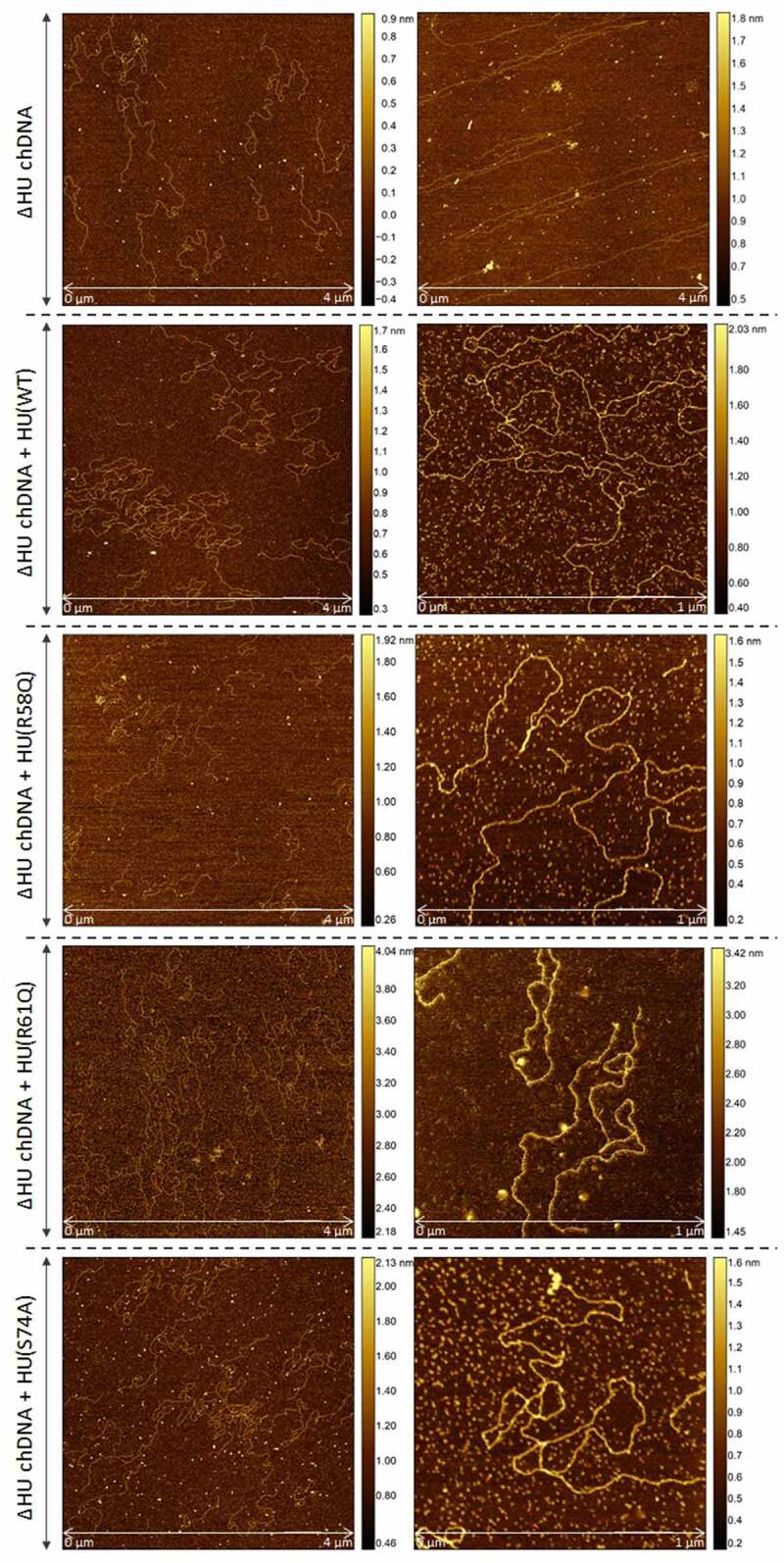
Survival of BALB/c mice upon infection of *F. tularensis*. The dose of 10^2^ CFU of WT strain FSC200 or mutant strains FSC200/HU_HA/R58Q, FSC200/HU_HA/R61Q, FSC200/HU_HA/S74A was administered (s.C.). Mice were observed daily for the following 30 days. All mice infected with WT strain FSC200 died within 7 days post infection whereas all mice infected with mutant strains survived. On day 30, these mice were challenged by infection with FSC200 strain (the dose of 3 × 10^2^ CFU/mouse) to study the ability of corresponding mutant strains to elicit protective immunity against WT strain. 90 % or 80 % of mice immunized with FSC200/HU_HA/R61Q or FSC200/HU_HA/S74A respectively and 100 % of mice immunized with FSC200/HU_HA/R58Q strain survived the WT challenge.

Further, we were interested in whether selected mutated HU protein amino acids have any influence on the relaxation or compaction of the chDNA. WT and mutant forms of HU protein were added to ΔHU chDNA to test whether they can restore the structure of ΔHU chDNA (length and compaction) to that of WT strain. The resulting changes in chDNA structure were visualized by AFM. As expected, we observed differences in the length of chDNA depending upon the HU protein mutant variant used (Figure 9). HU protein with a mutation in R58 was not able to compact ΔHU chDNA. Indeed, its length seemed to be even greater. On the other hand, when using R61Q and S74A mutant proteins the length of ΔHU chDNA shortened comparably to that of WT strain (Figure 8a, Figure 9 , and Figure 10). The differences were statistically significant, indicating that these amino acids are less important for DNA shape alteration than is R58. With this experiment, we confirmed the importance of R58 for chDNA compaction.

### *R58, R61, and S74 of the HU protein play crucial roles in FSC200* in vitro *proliferation*

**Figure 11.**
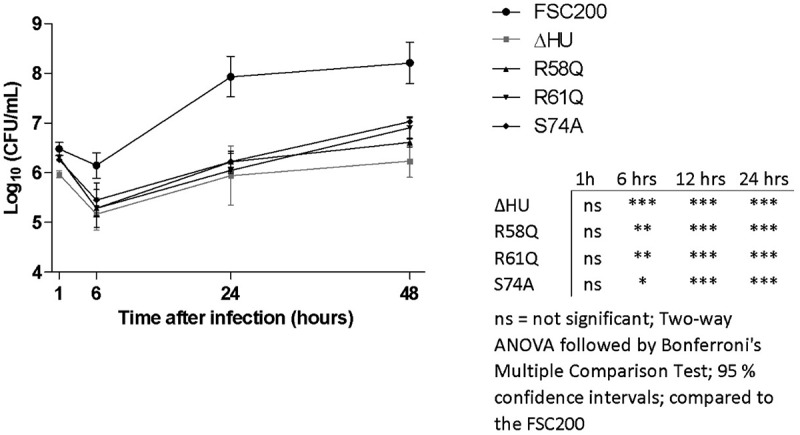
The effect of HU protein on the length and shape of *Francisella* chDNA. (A) Comparison of lengths of chDNA isolated from WT and ΔHU strains measured after AFM visualization revealed significant differences. The absence of HU protein caused an extension of ΔHU strain chDNA of about 30 %. Student´s *t*-test P-value <0.05* (B) AFM images of chDNA from WT and ΔHU strains. The chDNA from the WT strain showed an ordinary compact structure whereas chDNA from the ΔHU strain was more relaxed and long straight strands were observed. For both strains two images of chDNA obtained from different AFM scans are displayed in row. The height profile scale is shown on the right.

We previously have shown that mutant strain with deletion of the *hupB* gene was attenuated *in vitro* [20]. In order to uncover the necessity of R58, R61, or S74 for intra-macrophage replication, *in vitro* proliferation assays were performed. We compared FSC200/HU_HA/R58Q, FSC200/HU_HA/R61Q, and FSC200/HU_HA/S74A strains with deletion mutant strain FSC200/ΔHU and WT strain FSC200 (Figure 11). Surprisingly, all three amino acid alterations showed the importance in FSC200 *in vitro* proliferation and their substitution led to decreased ability of bacteria to survive and replicate inside bone marrow-derived macrophages (BMMs). All mutant strains (R58Q, R61Q, and S74A) multiplied in manners comparable with that of the deletion mutant strain FSC200/ΔHU, suggesting these amino acids are necessary for the proper functioning of the HU protein and have an influence on the overall virulence of FSC200 *in vitro*.

### Mutations in R58, R61, and S74 of the HU protein attenuate the virulence of FSC200 in mice and elicit protective immunity against WT challenge

**Figure 12.**
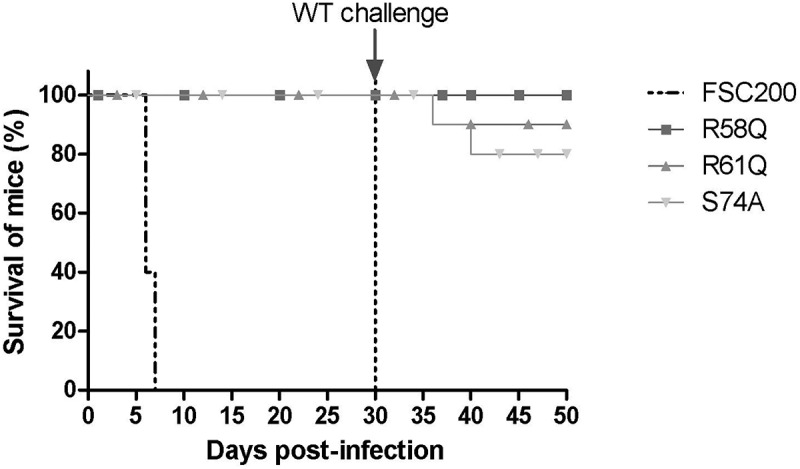
Survival of BALB/c mice upon infection of F. tularensis. The dose of 10^2^ CFU of WT strain FSC200 or mutant strains FSC200/HU_HA/R58Q, FSC200/HU_HA/R61Q, FSC200/HU_HA/S74A was administered (s.c.). Mice were observed daily for the following 30 days. All mice infected with WT strain FSC200 died within 7 days post infection whereas all mice infected with mutant strains survived. On day 30, these mice were challenged by infection with FSC200 strain (the dose of 3 x 10^2^ CFU/mouse) to study the ability of corresponding mutant strains to elicit protective immunity against WT strain. 90 % or 80 % of mice immunized with FSC200/HU_HA/R61Q or FSC200/HU_HA/S74A respectively and 100 % of mice immunized with FSC200/HU_HA/R58Q strain survived the WT challenge.

Further, we investigated if mutations in R58, R61, and S74 have any influence on FSC200 virulence *in vivo*. Mutant strains with substituted R58, R61, and S74 were administered to BALB/c mice at a 10^2^ CFU/mouse dose of infection, and the survival of mice was compared to that of mice infected with WT strain FSC200. All mice in the control group died within 7 days post-infection, whereas all mice infected with mutant strains survived (Figure 12). Further, we investigated the ability of mutant strains to elicit protective immunity against the WT challenge. Surviving mice were reinfected on day 30 post infection with FSC200 strain at a 10^2^ CFU/mouse dose of infection. Previous experiments had shown that the spleens of surviving mice were free of bacteria before this next challenge (data not shown). Mice were observed, and possible deaths were recorded. All mice immunized with the FSC200/HU_HA/R58Q survived the WT challenge. Only one mouse died in a group of mice immunized with FSC200/HU_HA/R61Q (on day 6 post-infection), and two mice did not survive the WT challenge after immunization with FSC200/HU_HA/S74A. This data showed the importance of R58, R61, and S74 of the HU protein in the virulence of FSC200 and suggest that mutated strain FSC200/HU_HA/R58Q has potential to become an effective vaccine strain against tularaemia.

## Discussion

HU protein is an essential component of *F. tularensis* subsp. *holarctica* FSC200 virulence [20]. Given that HU protein has been shown to bind DNA and is thought to function as a transcription factor, the questions arise as to in which areas and in which ways does it act and what does it regulate. Revealing the molecular basis of the HU protein function could aid us in understanding the virulence mechanisms of FSC200 and of the entire genus *Francisella* generally. HU protein binds to both double-stranded and single-stranded DNA, RNA, and all their hybrid forms [39]. Despite that the HU protein binding motif has not yet been found and that the protein is thought to be sequence non-specific, it shows a preference for abnormal structures [15]. Another study has shown that *E. coli* HU-β protein favoured the A/T rich binding region [16]. The mechanism of binding and bending DNA by HU protein is still unclear. Nevertheless, some conserved amino acids have been proven to be essential for DNA binding (e.g. proline residues) [40]. These are conserved among all homologs and have a key role in DNA-intercalation. DNA-intercalating proline is a part of the conserved RNP motif (R61-N62-P63 in FSC200), and arginine included in this sequence motif has a role no less important in the DNA binding function of HU protein. This arginine forms hydrogen bonds to the DNA in the case of integration host factor (IHF, an HU homolog) and it is also essential for generating crystal complex of the *Anabaena* HU protein [41]. These are not the only conserved amino acids that have been reported as important for the interaction of HU protein with DNA. Here, we focused on R58, R61, and S74. R61 is a part of the conserved RNP motif. R58 and R61 are analogous to corresponding DNA-interacting amino acids in *Mycobacterium tuberculosis* [28] and *Bacillus stearothermophilus* [42]. S74 corresponds to T74 in *M. tuberculosis*, where T74 is phosphorylated and this phosphorylation has been shown important for interaction with DNA [43].

In order to determine HU protein binding sites in the FSC200 genome, we decided to perform the ChIP-seq experiment. Evaluation criteria were fulfilled by 261 HU protein binding sites during the standard growth conditions in the early growth phase of bacteria and by 105 binding sites during the oxidative stress growth conditions in the early growth phase. No overlap between detected binding sites was observed (Table S2, available at: https://docs.google.com/spreadsheets/d/1wAT9OcUTomvDJudhI5963gF-rS3JwsZp/edit#gid=537570250), suggesting that HU protein regulates different parts of DNA depending upon signals from the outer environment. This is suggested, too, by the finding of two different binding motifs. In a study by Oberto et al. [18], where they mapped the HU regulon using a genome-wide *in silico* transcriptomic approach, 353 genes regulated by *E. coli* HU protein were found. Similarly to our findings, they observed the *E. coli* HU regulon to be composed of genes involved in anaerobiosis, acid stress, high osmolarity, and SOS induction [18]. Several genes detected in the FSC200 HU regulon overlapped with those from the *E. coli* HU regulon (highlighted in bold in Table S2 – standard peaks, available at: https://docs.google.com/spreadsheets/d/1wAT9OcUTomvDJudhI5963gF-rS3JwsZp/edit#gid=537570250). For example, genes belonging to the operons of *clpB, dna, gal, lys, pur, put*, and *rec* were detected under standard growth conditions. Several genes detected as possible binding sites of FSC200 HU protein during stress growth conditions belong to the same operon as do those that were also found in the *E. coli* HU regulon (highlighted in bold in Table S2 – stressed peaks). These include, for example, *atp, clpB, fum, pur, pyr*, and *suc*. The number of HU protein regulation sites identified can differ depending upon the method used, quality of the experiment, bacterial species, and growth conditions. HU protein should be considered as a global regulator with many binding sites throughout the genome.

Because detection of binding sites during the late exponential growth phase failed, we presupposed the amount of HU protein in the cell during the late exponential phase to be enormous, thereby causing the signal to be blurred and so no significant peaks could be determined in the data analysis. Binding motifs (Figure 2) were identified in all isolated DNA sequences analysed by the ChIP-seq approach. Subsequently, we were interested in how many times these motifs are represented in the whole FSC200 genome. Using FIMO software, we identified 20,399 standard and 14,882 stressed binding motifs throughout the whole genome (Table S3, available at: https://docs.google.com/spreadsheets/d/1Qm5AVHQB9DMnKIo7xzYQ6rhm8JZEP-z1/edit#gid=362571185), suggesting the possibility for high frequency of the HU protein binding. In order to display the frequency of bound HU proteins, we used AFM to visualize parts of DNA that interact with HU protein. Although we are able to see proteins bound to parts of the DNA (Figure 3, white dots), we cannot say for sure (at least in the case of standard growth conditions) that the frequency of HU protein binding to the DNA is significantly higher in the late exponential growth phase than during the early growth phase.

In our previous study [20], we described PigR as a protein with a significantly reduced expression on protein and transcriptional level in HU deletion mutant strain, as well, suggesting a role of HU protein in *pigR* gene expression. Despite that we did not detect the DNA region upstream of the *pigR* gene in the ChIP-seq analysis data, we confirmed the presence of HU protein binding sites and the ability of HU protein also to bind this region (Figure 4), suggesting that interaction of HU protein and DNA sequences around *pigR* promoter could occur. Using the EMSA method, we observed the ability of HU protein to bind the region 477 bp long upstream of the *pigR* gene. Identification of both standard and stressed binding motifs in this one particular sequence suggests that the regulation of virulence gene transcription factor PigR expression through HU protein might exist and might be dependent upon the conditions to which FSC200 is presently exposed.

The bending of DNA by transcription factors is an important part of gene expression and initiation of DNA replication. Among the important effects of HU protein on DNA shape is its ability to introduce negative supercoiling of DNA molecule in the presence of topoisomerase I in *E. coli* [44]. We studied the importance of three key amino acids in HU protein functioning: R58, R61, and S74. These three conserved amino acids have been reported as important for DNA binding capacity or negative supercoiling induction in other bacteria [28,42,43]. Greater importance for FSC200 HU protein function was observed in the case of R58 than of either R61 or S74. We showed that mutation in R58 led to a defect in HU protein’s capacity for DNA binding at the sequence upstream of the *pigR* gene (Figure 5a). Using RT-PCR, the role of R58 in *pigR* expression was definitely proven (Figure 6). Next, we confirmed that FSC200 HU protein can bind to a part of the *clpB* gene, the sequence that was identified in ChIP-seq analysis, where R61 showed greater importance in HU–*clpB*DNA complexes generation than did R58 (Figure 5b, HU–DNA complexes highlighted by red arrows). Nevertheless, R58 might be involved in HU–*clpB*DNA complex formation, as well, inasmuch as the amount of DNA in the complex is decreased in comparison to that in WT and S74, thus indicating different reaction kinetics. R58 also showed its importance in connection with the reduced ability to introduce negative supercoiling of DNA (Figure 7). Despite R61’s being a part of the conserved RNP motif that is important for the DNA binding capacity of HU protein [28,41], we observed greater importance of conserved R58 than of R61 in all experimental assays except HU–*clpB*DNA complexes formation in FSC200 HU protein. We also demonstrated that HU protein is responsible for wrapping the chDNA (Figure 9), similarly to as seen in other bacteria [45–47], and we showed the necessity of R58 (rather than of R61 and S74) in the ability of HU protein to change chDNA shape and length (Figure 9). Inasmuch as the identified binding motifs of the HU protein (Figure 2) are rather variable, we suggest the importance of R58 and R61 can fluctuate in the recognition of different HU protein DNA binding sites. Generally, chemical properties of arginine, such as positive charge, allow it to interact with negatively charged molecules such as DNA, and, due to the great diversity of post-translational modifications of arginine in bacteria [48], this may be an indispensable component of the virulence factor HU protein in FSC200. Finally, we showed the importance of three amino acids of the HU protein in FSC200 virulence both *in vitro* and *in vivo*. Surprisingly, we demonstrated that bacterial strains carrying mutated HU protein in R58, R61, and S74 are all attenuated during intracellular replication inside BMMs in manners similar to that seen for the deletion mutant strain lacking the HU protein (Figure 11). Although the HU_HA/R61Q and especially the HU_HA/S74A proteins – in contrast to HU_HA/R58Q – show certain DNA binding capacity during *in vitro* experiments with purified proteins, all three mutant strains are defective in intramacrophage replication. This suggests another mode of HU protein action to be involved in this process. Disruption of HU protein function and thus of overall bacterial virulence also manifested when testing the ability of mutant strains to develop tularaemia in a mouse model. Not only did the mice not die when infected with the mutant strains, but these mice also were able to withstand subsequent WT challenge. The most effective of these was the mutant strain with substitution of R58 (Figure 12), suggesting a possible vaccine strain against tularaemia development. In the light of our findings, we can see that HU protein offers substantial potential for further studies focused on *Francisella* vaccine research or to be a target molecule for generating an anti-*Francisella* agent or even additional antimicrobials more generally.

## Supplementary Material

Supplemental Material

## Data Availability

The data that support the findings of this study are openly available in this manuscript and supplementary files.
