# Supplementary material for: Arginine 58 is indispensable for proper function of the Francisella tularensis subsp. holarctica FSC200 HU protein, and its substitution alters virulence and mediates immunity against wild-type strain
Source: Virulence. 2022 Oct 17;13(1):1790–809. doi: 10.1080/21505594.2022.2132729 (PMC9578482; doi:10.1080/21505594.2022.2132729)
Supplement: Supplemental Material [file KVIR_A_2132729_SM0729.zip › supplementary/Table_S1_primers.docx]

| **Primer** | **Sequence** | **Reverse/Forward** | **Application** |
| --- | --- | --- | --- |
| pKK_0886_F | 5´AAACATATGAACAAGAGTGAATTAGTAAG3´ | F | Construction of FSC200/HU_HA strain. Contains NdeI site (underlined). |
| pKK_0886_R_HA | 5´AAAGAGCTCTTA**AGCGTAATCTGGAACATCGTATGGGTA**TTTTACAGCGTCTTTAAGACC3´ | R | Construction of FSC200/HU_HA strain. Contains SacI site (underlined) and HA tag sequence (bold). |
| R58Q_sense | 5´TACTTTTCAGGTAAAAGAAAGAAGTCCTCAAGAGGGTAGAAACCCAAAG3´ | F | Construction of FSC200/HU_HA/R58Q strain. |
| R58Q_antisense | 5´CTTTGGGTTTCTACCCTCTTGAGGACTTCTTTCTTTTACCTGAAAAGTA3´ | R | Construction of FSC200/HU_HA/R58Q strain. |
| R61Q_sense | 5´GAAAGAAGTCCTAGAGAGGGTCAAAACCCAAAGACTGGTGAAAC3´ | F | Construction of FSC200/HU_HA/R61Q strain. |
| R61Q_antisense | 5´GTTTCACCAGTCTTTGGGTTTTGACCCTCTCTAGGACTTCTTTC3´ | R | Construction of FSC200/HU_HA/R61Q strain. |
| S74A_sense | 5´TGAAACTATTAAGATCCCTGCTGCTAAAGTTCCTAGCTTTAAAGC3´ | F | Construction of FSC200/HU_HA/S74A strain. |
| S74A_antisense | 5´GCTTTAAAGCTAGGAACTTTAGCAGCAGGGATCTTAATAGTTTCA3´ | R | Construction of FSC200/HU_HA/S74A strain. |
| PigR_F | 5´ATGGCGAATCAATATTCTGGAA3´ | F | RT-PCR |
| PigR_R | 5´CAGTCAAGATTTAGCTTTGATTA3´ | R | RT-PCR |
| RpoA_F | 5´GTGAGTAATAATAATTCAAAACTG3´ | F | RT-PCR |
| RpoA_R | 5´TTATTTTCCTTCAACTAGCTCTC3´ | R | RT-PCR |
| ClpB_F | 5´CAAGAAGGTAATGTAATTCTCT3´ | F | Amplification of part of the *clpB* gene for EMSA |
| ClpB_R | 5´GTCCTTTATACTCGGAGTCTA3´ | R | Amplification of part of the *clpB* gene for EMSA |
| Up_PigR_F | 5´TTC AAT GTG TTT TGT TAA TTT TCT C3´ | F | Amplification of 477bp sequence upstream of the *pigR* gene for EMSA |
| Up_PigR_R | 5´TAT AAC GAG TCT CCT TTA ATT A3´ | R | Amplification of 477bp sequence upstream of the *pigR* gene for EMSA |
