# Supplementary figures and images for: Arginine 58 is indispensable for proper function of the Francisella tularensis subsp. holarctica FSC200 HU protein, and its substitution alters virulence and mediates immunity against wild-type strain
Source: Virulence. 2022 Oct 17;13(1):1790–809. doi: 10.1080/21505594.2022.2132729 (PMC9578482; doi:10.1080/21505594.2022.2132729)

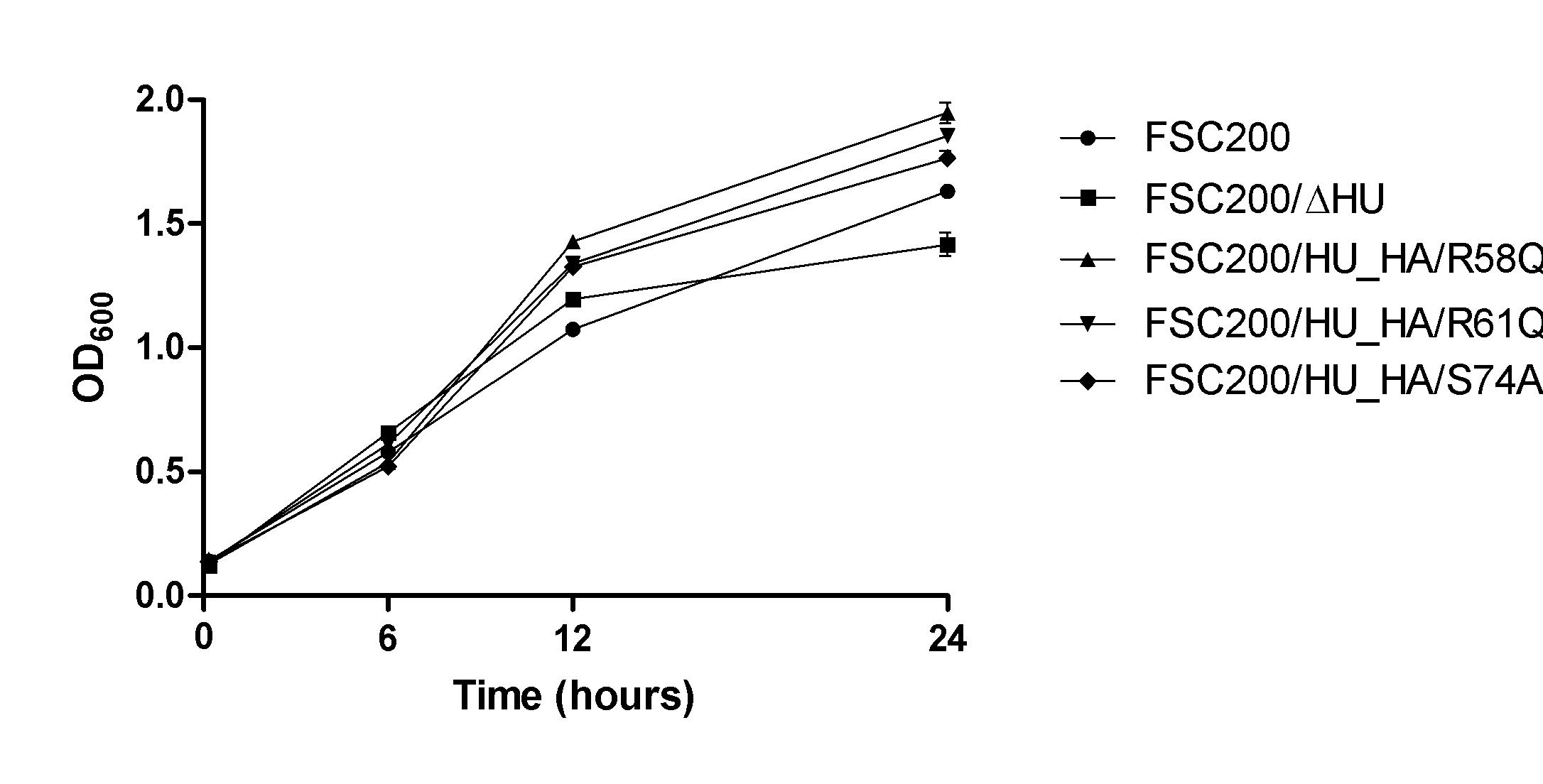

Supplement: Supplemental Material [file KVIR_A_2132729_SM0729.zip › supplementary/Figure_S1_HU_mutants_growth_curves.jpg]
